# Short-Term Effects over Time of Endotracheal Suctioning on Very Low Birth Weight Premature Infants with RDS

**DOI:** 10.3390/children12070808

**Published:** 2025-06-20

**Authors:** Ernetas Virsilas, Arunas Valiulis, Arunas Liubsys

**Affiliations:** Clinic of Children’s Diseases, Institute of Clinical Medicine, Faculty of Medicine, Vilnius University, LT-03101 Vilnius, Lithuaniaarunas.liubsys@santa.lt (A.L.)

**Keywords:** respiratory distress syndrome, mechanical ventilation, electrical impedance tomography, preterm newborns, endotracheal suction

## Abstract

Background: Respiratory distress syndrome (RDS) is a frequent cause of invasive respiratory support. Our study aims to assess end-expiratory lung impedance (EELZ) and DeltaZ changes post-suction using electrical impedance tomography. Methods: Very low birth weight infants with gestational ages less than 32 weeks under conventional mechanical ventilation with an open endotracheal suction system were included in this study. Data was evaluated at four time periods: immediately after the completion of suctioning and at 1, 5 and 10 min marks post-suction. Results: Sixteen patients participated in this study, during which a total of 31 suctioning events were recorded. There were no significant hypoxemic events during the analyzed timeframe. Over a 10 min period following suction, there was a consistent change in EELZ and DeltaZ, with EELZ decreasing and DeltaZ increasing accordingly (*p* < 0.001). Conclusions: Our study demonstrated that EELZ and DeltaZ changes persist even 10 min after suctioning using an open endotracheal suction system.

## 1. Introduction

Pediatric and neonatal intensive care patients often need intubation due to severe, life-threatening conditions. Since intubated patients are unable to remove excess secretions due to an ineffective cough reflex, thick secretions and/or sedation, maintaining airway potency in intubated patients is an important aspect of intensive care [1]. There are two notable suctioning systems: an open tracheal suction system (OTSS) and a closed tracheal suction system (CTSS). An OTSS requires disconnecting the patient from ventilation before performing the procedure, while a CTSS allows to partially continue ventilation while suctioning. It is believed that a CTSS offers several advantages, such as fewer hypoxemic events, reduced exposure of secretions to the staff and possibly lower ventilator-associated pneumonia rates [2]. However, it also increases airway resistance up to 22% and augments unventilated “dead space” [3]. These concerns are typically not significant issues in adults and older children. However, in neonates, especially very low birth weight infants, they contribute a substantial portion to the unventilated circuit and can account for up to 30% of tidal volumes. We evaluated end-expiratory lung impedance (EELZ) and DeltaZ in intubated patients with RDS after using an OTSS, both of which closely correlate to end-expiratory lung volume (EELV) and tidal volume (TV), respectively.

## 2. Methods

### 2.1. Trial Design

The data were obtained from a prospective cohort of 16 infants under conventional mechanical ventilation treated in a tertiary-level NICU. This study was approved by the regional ethics committee and registered prospectively at clinicaltrials.gov (reg. no. NCT04542096). Informed parental consent was obtained before enrolment. Very low birth weight (VLBW) (<1500 g) infants with gestational ages (GAs) less than 32 weeks and a need for invasive respiratory support using conventional ventilation were enrolled. The exclusion criteria included skin damage or abrasions on the chest at the EIT belt attachment site and significant thoracic deformities that could affect EIT readings.

### 2.2. Participants

Anthropometric and demographic data were obtained after enrolment. EIT data was recorded using an electrical impedance tomography device (Enlight 1800, Timpel, São Paulo, Brazil) at a sampling rate of 50 frames per second using neonatal belts with 16 electrodes. All patients were suctioned on an on-demand basis. Preoxygenation was routinely employed for hypoxemia prevention and was defined as a 20% increase in FiO_2_ above baseline for 90 s. All included patients had a stable oxygen requirement of less than 0.3 FiO₂ for at least 12 h prior to inclusion. No comorbidities related to prematurity-associated RDS were present.

### 2.3. Data Collection and Analysis

For EIT data collection, neonatal belts equipped with 16 evenly spaced electrodes were placed circumferentially around the thorax at the nipple level. Data analysis was performed off-line with Timpel Medical’s (São Paulo, Brazil) custom-designed analysis software. A period of 10 consecutive minutes was used for the analysis of the different periods at 0 (immediately after the completion of suctioning), 1, 5 and 10 min post-suctioning. EELZ and DeltaZ were calculated, adjusted for body weight and expressed as arbitrary units per kilogram of body weight (AU/kg).

### 2.4. Statistical Methods

Depending on their distribution, data were expressed as mean ± SD or as median with interquartile ranges. Normality was assessed by using the Shapiro–Wilk test. A linear mixed-effect model for repeated measures was used for EELZ and DeltaZ analysis, with an assessment to account for multiple breaths from each infant. To mitigate the issue of multiple comparisons in the analysis of the mixed-effects model, we utilized the Bonferroni correction. A *p*-value of less than 0.05 was considered to be statistically significant. Statistical analysis was performed using R statistical software (R Foundation for Statistical Computing, Vienna, Austria; version 4.1.3).

## 3. Results

**Figure 1:**
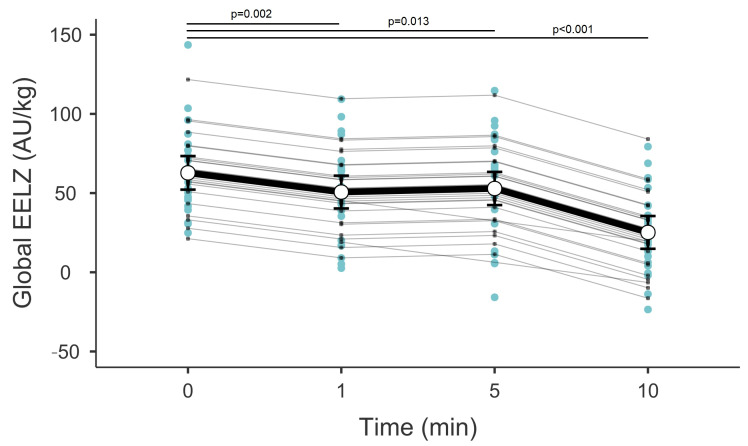
EELZ over time post-suctioning. Error bars represent the mean with 95% confidence intervals. Blue dots represent multiple measurements at each time point from all patients. Grey connecting lines represent random effects. AU—arbitrary units.

**Figure 2:**
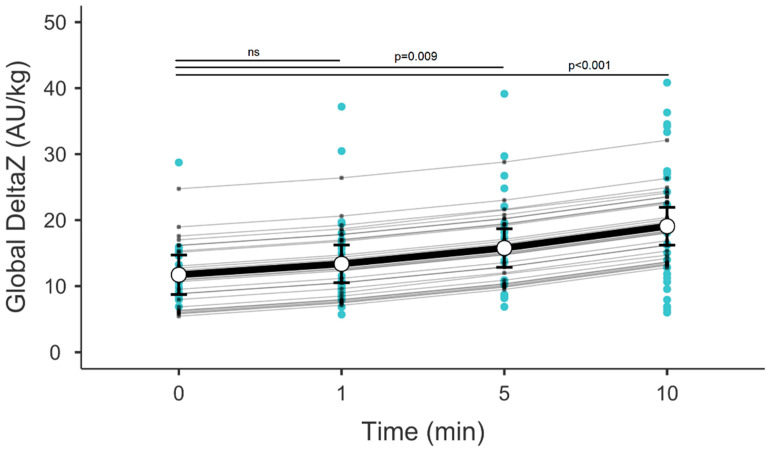
DeltaZ over time post-suctioning. Error bars represent the mean with 95% confidence intervals. Blue dots represent multiple measurements at each time point from all patients. Grey connecting lines represent random effects. AU—arbitrary units.

A total of 16 patients under mechanical ventilation were enrolled, and a total of 31 suctioning events were documented. Patient anthropometric and demographic characteristics are outlined in Table 1. There were no significant desaturation events in the cohort. Over a 10 min period following suction, there was a consistent change in EELZ and DeltaZ, with EELZ decreasing and DeltaZ increasing accordingly (*p* < 0.001, Figure 1 and Figure 2). While EELZ appeared to reach a stable point at the 5 min, this effect proved to be transient, as there was still a continuous loss of EELZ at the 10 min mark. DeltaZ exhibited a continuous increase throughout the measured periods without variations.

## 4. Discussion

Respiratory distress syndrome frequently impacts premature infants with low birth weight, leading many to require intubation [4]. To maintain airway potency, airway management includes suctioning as needed. Sedation and endotracheal cuffs are seldom used for neonates receiving invasive respiratory support, often resulting in higher demand for the procedure. The most frequent short-term complications are hypoxemia and bradycardia during suctioning, with the former typically being amended by preoxygenation. Bradycardic events are also rarer, since most recommendations agree that deep and/or prolonged suctioning is not beneficial and possibly harmful [5,6].

In our study, there were no significant hypoxemic events. There was only one case of marked bradycardia. However, it should be noted that, unlike some previous studies, smaller heart rate fluctuations were not collected [7,8]. There was a significant ongoing loss of EELZ (which strongly correlates with end-expiratory lung volume (EELV)) even at the 10 min mark post-suction [9]. While certain studies also reported a reduced lung volume after suction [10], other studies found that this volume is restored relatively quickly, which is in contrast with our findings [11]. However, the aforementioned studies followed a shorter post-suction period, potentially obscuring prolonged effects. Secondly, observations might be due to the older patient population (infants and young children) and different primary diagnoses. In contrast to localized pulmonary conditions like pneumonia, neonatal RDS is a highly uniform disease that typically affects the entire lung; thus, the loss of positive end-expiratory pressure might have a detrimental impact on large areas of the lung. Finally, the utilization of an OTSS likely contributed to the findings, as specific studies have shown that while suctioning can lead to significant lung impedance reduction, the recovery of these losses tends to be notably faster with a CTSS, even though EELZ may not always return to its pre-suction level [12]. This is also supported by findings comparing the OTSS and CTSS in neonates, even though definitive conclusions regarding the preferred method of suctioning still cannot be drawn for patients under conventional ventilation [13].

DeltaZ (which strongly correlates with tidal volume (TV)) increased over time post-suctioning [9]. This might be due to the removal of secretions following the procedure, allowing the patient to inhale larger volumes. Our findings are in agreement with other research that showed that TV gain extends over time after suctioning [14]. It could also be due to a larger amount of secretions removed with an OTSS compared to a CTSS [15]. Nevertheless, not all studies replicated the aforementioned findings, and evidence of the OTSS’s superior efficacy in secretion removal remains inconclusive [16].

We must acknowledge several limitations of our study. The most significant is the absence of a direct head-to-head comparison with a CTSS, which could affect the interpretation of our results. Although previous studies have evaluated a CTSS and its effects, some even using EIT, incorporating it would have offered a more robust comparison. Additionally, even though we thought we selected an adequately large timeframe of ten minutes, extending this period might have yielded additional information.

However, this study presents several strengths. In particular, it is one of the few studies to evaluate open endotracheal suctioning, a practice that has become largely obsolete due to shifts in clinical management and hospital policies, despite the lack of clear evidence supporting the superiority of a CTSS. Moreover, we utilized EIT to estimate the effects of suctioning on EELV and TV, as direct assessment using flow sensors during the use of an OTSS is unfeasible due to sensor loss, and conventional indirect measures like PaO_2_ or FiO_2_ fail to capture the lung dynamics, which we sought to address.

## 5. Conclusions

Our study demonstrated that EELZ and DeltaZ changes persist longer after suctioning using an OTSS under conventional mechanical ventilation than previously recorded, with a marked loss of EELZ even 10 minutes post-suction. It is advisable to avoid open suction systems in patients with compromised lung recruitment, especially in those who are medically unstable, given the potential for prolonged adverse effects.

## Figures and Tables

**Table 1:** Patient anthropometric and demographic data. The data are expressed as mean (±SD) or median (IQR). FiO_2_—fraction of inspired oxygen requirement before suctioning; SpO_2_—peripheral oxygen saturation; BPM—beats per minute.

Variable		Neonates (n = 16) Suctioning Events (n = 31)
Postnatal age (days)	9.96 (8.1)	Postnatal age (days)
Gestational age (weeks)	27.27 (±2.6)	Gestational age (weeks)
Birth weight (g)	1016.7 (±283)	Birth weight (g)
Weight at the time of study (g)	1066.4 (±223)	Weight at the time of study (g)
Male (n)	8 (50%)	Male (n)
Small for gestational age (n)	1 (6.25%)	Small for gestational age (n)
Surfactant administration (n)	15 (93.75%)	Surfactant administration (n)
FiO_2_	0.24 (0.07)	FiO_2_
Respiratory rate (breaths/min)	55.3 (±8.4)	Respiratory rate (breaths/min)
SpO_2_	90.4 3 (±4.22)	SpO_2_
Significant bradycardic events (<100 BPM) (n)	1 (3.22%)	Significant bradycardic events (<100 BPM) (n)

## Data Availability

The datasets used and/or analyzed during the current study are available from the corresponding author on reasonable request.
